# Acute Kidney Injury Secondary to NSAID Diagnosed on 99mTc MDP Bone Scan

**DOI:** 10.4274/Mirt.95

**Published:** 2013-08-01

**Authors:** Özhan Özdoğan, Nazlı Pınar Karahan, Sülen Sarıoğlu, Hatice Durak

**Affiliations:** 1 Dokuz Eylül University Faculty of Medicine, Department of Nuclear Medicine, İzmir, Turkey; 2 Dokuz Eylül University Faculty of Medicine, Department of Pathology, İzmir, Turkey

**Keywords:** Acute kidney injury, 99mTc-MDP, anti-inflammatory agents, non-steroidal

## Abstract

A bone scan with 99mTc MDP was obtained to rule out the presence of micro fractures in a patient with the diagnosis of idiopathic osteoporosis. There was not any sign of micro fractures, but interestingly, both kidneys were diffusely very active. A differential diagnosis of acute kidney injury secondary to the use of nonsteroidal anti-inflammatory drug was made and reported after elimination of other clinical situations. The renal functions obtained following bone scan were impaired. The anti-inflammatory drug was discontinued. The renal functions were recovered starting with the following day.

**Conflict of interest:**None declared.

## INTRODUCTION

Bone scintigraphy is one of the frequently used imaging tests in nuclear medicine. It is applied as a screening test for some oncologic and non-oncologic diseases. In this case report we present a patient with non-steroidal anti-inflammatory drug (NSAID) induced acute kidney injury which was diagnosed by bone scintigraphy.

## CASE REPORT

**Figure 1:**
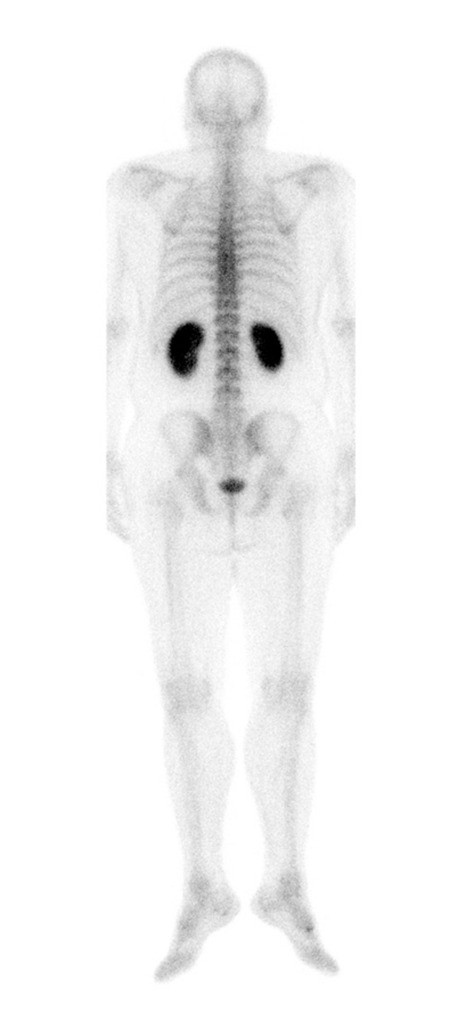
Posterior projection of whole body bone scan. A marked and diffuse increase in renal uptake is observed

A 50 year old male with the diagnosis of idiopathic osteoporosis was experiencing pain especially in the thoracolumbar area. He was taking NSAIDs for pain relief for the last few months. He was referred for bone scintigraphy upon exacerbation of his symptoms to rule out the possible micro fractures. The bone scan was obtained at 4th hour following the injection of 925 MBq of technetium 99m methylene diphosphonate (99mTc MDP). The images were obtained with low energy high resolution collimator with a 20 % window set at a 140 keV energy peak. The bed speed was 9 cm/min for continuous acquisition. There were not any significant focal activities indicating micro fractures in the skeleton. But, interestingly diffuse 99mTc MDP uptake was noticed bilaterally in the kidneys (Figure 1). 

The patient anamnesis and the patient files were investigated for the possible differential diagnosis of the observed diffuse renal uptake. There was not a history of an oncologic or chronic disease. There was not any history of chemotherapy, radiation therapy or antibiotherapy. There were not any complaints of urinary tract obstruction or symptoms of urinary tract infection. The patient was normotensive. The only medication the patient intakes was diclofenac (75 mg, 2x1 po.) for some months. So, the probability of acute kidney injury as the reason for the bilateral diffuse renal uptake of 99mTc MDP was reported. The intake of the NSAID was also noted as the possible reason for the acute kidney injury (AKI). 

Renal function tests and urine microscopy were ordered by the clinician. The blood urea nitrogen (BUN) and the creatinine values were found to be high (Table 1). On clinical evaluation, there was not any known underlying renal disease and there were not any signs and symptoms of dehydration to explain the increased values detected on renal function tests. The patient was normocalcemic. On urine microscopy, squamous epithelial cells, some reactive urothelial cells and a few cellular casts were observed. Some apoptotic cells were also observed. There was not any sign of inflammation and eosinophilia on urine microscopy. Although the urine microscopy results were most probably related with acute tubular injury, the possibility of tubulointerstitial nephritis could not be ruled out with the given findings. The therapy with the NSAID was discontinued. The BUN and creatinine values dropped gradually on the following days (Table 1) and the renal function tests returned to normal limits upon resolution of the tubular injury. The final clinical diagnosis was nonsteroidal anti-inflammatory drug (NSAID) induced acute kidney injury.

**Literature Review and Discussion**

In the presented case we observed an abnormally and diffusely increased MDP uptake in both kidneys. The incidence of diffuse renal uptake on bone scintigraphy was reported to be less than 1 % (1). One of the proposed mechanisms for the diffuse symmetrical increase is the renal injury of any kind that acts by adversely affecting the renal secretory and glomerular functions (2) or by the production of intracellular calcium in ischemic kidney (2,3). The overload of iron, by altering the distribution of bone seeking agents and decreasing the renal excretion, was also proposed to cause diffuse renal uptake on bone scan (2,4). The literature was reviewed for the bilateral diffuse renal uptake of bone seeking agents and the causative factors (2,3,4,5,6,7,8,9,10,11,12,13,14,15) were given in a table (Table 2). In one case report, Watanabe and his coworkers confirmed an exercise induced ATN in a young patient with renal biopsy which was diagnosed on bone scan (15). 

The presented patient did not have any oncologic and chronic disease. So the effects of chemotherapeutics and antibiotics as well as the chronic diseases causing iron overload were excluded. He was normotensive and normocalcemic which made the diagnosis of metabolic and vascular pathologies unlikely. The most probable cause for the visualization of the hot kidneys was AKI secondary to the use of a NSAID. AKI is a sudden decrease of the renal function manifesting with an increase in BUN and serum creatinine. The causes of AKI is divided in 3 categories and given in the table (Table 3).

Ting and his coworkers described a case of AKI presented with diffuse renal uptake on bone scan secondary to use of significant amounts of NSAIDs (2). As far as we know this is the second case in the literature which demonstrates a diffuse and marked MDP accumulation in kidneys secondary to the use of NSAIDs. Interestingly, a patchy and multiple increased renal uptake on bone scan in addition to the diffuse uptake, secondary to the use of analgesics was also described in the literature (16). So the hot kidneys were described both in a diffuse or patchy form secondary to the use of analgesics. 

The medications causing renal failure act with several mechanisms. A hemodynamic renal failure may be caused by COX-2 selective inhibitors and ACE inhibitors/angiotensin II receptor blockers. Some antiviral drugs cause direct proximal tubular epithelial toxicity. Some other drugs like methotroxate and sulfonamides can cause ARF via deposition of crystals (crystal nephropathy) in the kidneys (17). The renal side effects of NSAIDs are very rare. NSAIDs, either they are selective or nonselective cyclooxygenase inhibitors, result in some acute effects (AKI, hypertension, edema, congestive heart failure, hyponatremia and hypokalaemia and tubulointerstitial nephritis). The chronic effects (nephritic syndrome, analgesic nephropathy, chronic papillary necrosis and urologic cancers) and acute papillary necrosis were only reported with nonselective COX inhibitors (17). 

Diclofenac is a selective COX-2 inhibitor. The risk of renal toxicity increases with the drug dose (18). The COX inhibitors decrease the synthesis of cyclooxygenase-derived prostaglandins and inhibit the major role of prostaglandins which is to preserve renal function whenever the physiologic processes were compromised (17). This is also true for the COX-2 selective inhibitors. The COX-2 selective inhibitors like diclofenac cause hemodynamic renal failure by reducing prostaglandins and decreasing renal blood flow and glomerular filtration rate (19). The selective COX inhibitors have equivalent nephrotoxicity as nonselective ones (17). The other mechanisms for the adverse renal effects caused by NSAIDs are the vasoconstrictor effect of thromboxane and reduction of prostacyclin synthesis (3,20). The renal side effects reverse in days, after discontinuing of the drugs (2). 

We did not perform a renal biopsy or a repeat bone scan for ethical reasons because the microscopy of the urine specimen was compatible with acute tubular injury and the renal functions returned to normal levels within a few days following the withdrawal of the drug. 

NSAIDs are widely used. Although rare, they may induce AKI which may proceed to chronic renal insufficiency if not recognized earlier. The nuclear medicine specialist must consider the possibility of NSAID induced AKI in cases with diffuse bilateral renal uptake of MDP.

## Figures and Tables

**Table 1:**
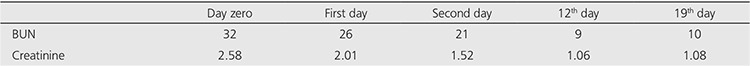
The BUN and creatinine levels on diagnosis and follow-up. Day zero is the last day on NSAID and day 1 is the first day without the drug. There was a gradual decrease of both BUN and creatinine values upon discontinuation of the drug on day 1 and day 2. On days 12 and 19 these values were completely in normal limits

**Table 2:**
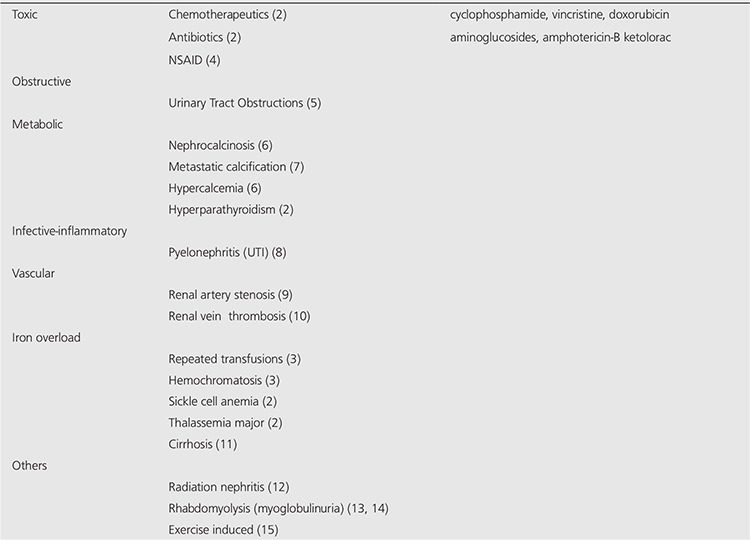
The differential diagnoses of bilateral diffuse renal uptake of bone seeking agents were given. The causative factors were found in the literature and a categorized table was constituted.

**Table 3:**
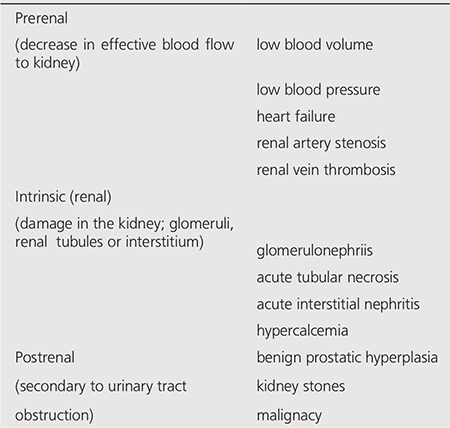
The causes of AKI
